# Efficacy and safety of transarterial chemoembolization combined with sintilimab and bevacizumab in hepatocellular carcinoma

**DOI:** 10.3389/fonc.2025.1739249

**Published:** 2026-01-12

**Authors:** Yi Wu, Miaoshen Yu, Tonggang Zhou, Yunfei Tian, Yusheng Song

**Affiliations:** 1 Department of Interventional Radiology, Ganzhou Hospital-Nanfang Hospital, Southern Medical University, Ganzhou, Jiangxi, China; 2 Department of Interventional Radiology, Ganzhou People’s Hospital, Ganzhou, Jiangxi, China

**Keywords:** bevacizumab biosimilar, efficacy, hepatocellular carcinoma, safety, sintilimab, transarterial chemoembolization

## Abstract

**Objective:**

Transarterial chemoembolization (TACE) is standard of care for patients with unresectable hepatocellular carcinoma (uHCC) that is amenable to embolization; however, its long-term prognosis is limited. Recently, TACE with systemic therapies showed meaningful improvement in clinical outcomes. This study aims to evaluate the real-world efficacy and safety of transarterial chemoembolization (TACE) combined with sintilimab and a bevacizumab biosimilar in the treatment of unresectable hepatocellular carcinoma (uHCC), which has been incorporated into the first-line treatment regimen in China.

**Methods:**

A retrospective analysis was conducted on 60 patients with uHCC who received TACE combined with sintilimab (200mg IV, q3w) and a bevacizumab biosimilar (15mg/kg IV, q3w) at Ganzhou People’s Hospital from August 2019 to June 2024, with follow-up until June 30, 2025. Overall survival (OS), progression-free survival (PFS), post-treatment tumor response, and treatment-related adverse events (tr-AEs) were recorded and analyzed. Use Kaplan-Meier for survival and Cox model for risk factors.

**Results:**

A total of 60 patients were enrolled in this study. The median OS was 24.0 months, and the median PFS was 13.0 months. According to the Response Evaluation Criteria in Solid Tumors (RECIST) v1.1, the best overall response rate (ORR) was 35.0%, and the disease control rate (DCR) was 91.7%. Based on the modified RECIST (mRECIST) criteria, the best ORR and DCR were 83.3% and 91.7%, respectively. Multivariate analysis identified macrovascular invasion as an independent risk factor for both OS and PFS (P < 0.05). All tr-AEs were manageable, and no treatment-related deaths occurred.

**Conclusion:**

TACE plus sintilimab-bevacizumab biosimilar has favorable efficacy and manageable safety in patients with uHCC, supporting its use as a treatment option in HCC.

## Introduction

Hepatocellular carcinoma (HCC) is the predominant primary malignancy of the liver, accounting for approximately 75-85% of all liver cancers. Worldwide, HCC ranks as the sixth most prevalent malignancy and the fourth leading cause of cancer-related mortality (1). Owing to its typically asymptomatic progression, most patients are diagnosed at intermediate or advanced stages, thereby missing the opportunity for potentially curative surgical treatment and consequently facing an unfavorable prognosis.

For patients who are unsuitable candidates for curative therapy, transarterial chemoembolization (TACE) remains a standard and widely adopted locoregional treatment approach (2). By obstructing the tumor’s arterial blood supply and inducing localized ischemic necrosis, TACE can effectively suppress tumor progression. However, this procedure also leads to the development of a hypoxic tumor microenvironment, which upregulates vascular endothelial growth factor (VEGF) expression and consequently promotes tumor recurrence and metastasis (3). As a result, the therapeutic efficacy of TACE monotherapy for HCC remains unsatisfactory (4).

In recent years, the advent of targeted immunotherapies has profoundly reshaped the therapeutic paradigm for unresectable HCC (5, 6). Combination regimens, such as atezolizumab plus bevacizumab, have been established as the standard first-line treatment for advanced HCC. The large multicenter Chinese clinical trial ORIENT-32 demonstrated that sintilimab combined with a bevacizumab biosimilar achieved significantly greater efficacy than sorafenib, resulting in higher overall response rate (ORR) and prolonged progression-free survival (PFS) (7). Based on these results, the sintilimab-bevacizumab biosimilar combination has been endorsed in clinical guidelines as a first-line systemic therapy.

Although the systemic efficacy of sintilimab in combination with a bevacizumab biosimilar has been well established, research exploring its combination with TACE in patients with HCC are still relatively scarce. Therefore, the present study aimed to investigate the efficacy and safety of TACE combined with sintilimab and a bevacizumab biosimilar in patients with uHCC, thereby providing additional clinical evidence to support therapeutic decision-making.

## Materials and methods

### Patients

Patients with uHCC who received TACE combined with sintilimab and a bevacizumab biosimilar at Ganzhou People’s Hospital between August 2019 to June 2024 were retrospectively enrolled. Inclusion criteria were as follows: (1) age ≥18 years; (2) clinically or histologically confirmed HCC; (3) an Eastern Cooperative Oncology Group (ECOG) performance status of 0-1; (4) Child-Pugh class A or B liver function; (5) at least one measurable lesion according to the modified Response Evaluation Criteria in Solid Tumors (mRECIST); (6) unable to perform radical resection due to oncological or surgical unresectability. Exclusion criteria were: (1) incomplete clinical data; (2) presence of severe cardiac, cerebrovascular, pulmonary, or renal disease; (3) concomitant untreated malignancies. This study was approved by the Ethics Committee of Ganzhou People’s Hospital (Ethical Approval No. PJB2025-355-01).

### Treatment methods

*TACE procedure*: Under local anesthesia, the Seldinger technique was used to insert a catheter via the femoral artery into the celiac or common hepatic artery for angiography. Superselective catheterization of the tumor-feeding artery was then performed. According to the tumor size, an appropriate dose of chemotherapeutic agents (20–40 mg lobaplatin and 20–40 mg epirubicin) was mixed with 10 mL of iodized oil for embolization. When the tumor vasculature was saturated and the portal venous branches around the lesion showed stasis, 300–500 μm polyvinyl alcohol (PVA) particles or microspheres were slowly injected until blood flow ceased, achieving complete embolization. Post-embolization angiography was performed to confirm satisfactory results. The catheter and sheath were then withdrawn, and the puncture site was compressed and bandaged to prevent bleeding. Postoperative monitoring included vital signs (heart rate, blood pressure) and general condition. After 24 hours, the bandage was removed, and the puncture site and limb mobility were examined. Hepatoprotective and symptomatic treatments were administered as needed. The interval between TACE sessions was determined based on imaging evaluations.

*Targeted and immunotherapy* Systemic therapy was initiated seven days after TACE. Sintilimab (200 mg) and bevacizumab (15 mg/kg) were administered through intravenous infusion, with each cycle repeated every three weeks. Dose reductions or treatment discontinuation was implemented according to the severity of treatment-related adverse events (tr-AEs). When deemed appropriate, patients were allowed to continue with either agent as monotherapy.

### Follow-up

Follow-up evaluations were initiated 4–6 weeks after TACE and subsequently carried out at three-week intervals. Each follow-up included laboratory assessments such as complete blood count, alpha-fetoprotein (AFP) level, liver function, renal function, and coagulation profile. Radiological evaluations—such as chest CT and contrast-enhanced CT or MRI of the upper abdomen—were performed every 2–3 months after TACE. Follow-up was maintained through June 30, 2025, in alignment with the survival data cutoff.

### Efficacy and safety analysis

The primary endpoint was overall survival (OS), defined as the time from treatment initiation to death from any cause or the end of follow-up. Secondary endpoints included PFS, ORR, disease control rate (DCR), and adverse events (AEs). PFS was defined as the time from treatment initiation to the first disease progression, death, or the end of follow-up. Treatment efficacy was evaluated according to mRECIST/RECIST v1.1 and categorized as complete response (CR), partial response (PR), stable disease (SD), or progressive disease (PD). ORR = (CR + PR)/total cases; DCR = (CR + PR + SD)/total cases. The severity of AEs was graded and recorded based on the Common Terminology Criteria for Adverse Events (CTCAE) version 5.0.

### Statistical analysis

All statistical analyses were performed using SPSS version 25.0 and R version 4.4.3. Cox proportional hazards regression analysis was applied to identify prognostic factors. Variables with P < 0.05 in univariate analysis were included in multivariate Cox regression. In multivariate analysis, P < 0.05 was considered statistically significant and indicative of an independent prognostic factor. OS and PFS were estimated using the Kaplan-Meier method, and survival curves were plotted. Waterfall plots of treatment response were generated using GraphPad Prism version 9.3.1.

## Results

### Baseline characteristics of patients

**Figure 1:**
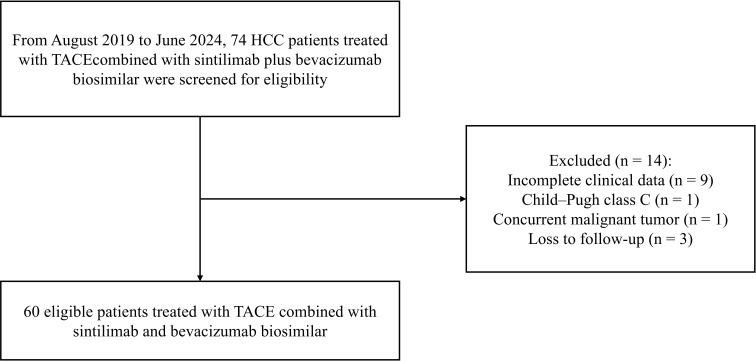
Flowchart of participant enrollment and inclusion.

The flowchart illustrating patient inclusion and exclusion criteria is presented in **Figure 1**. A total of 60 patients with uHCC who received a combination of sintilimab, bevacizumab biosimilar, and TACE were enrolled between August 2019 and June 2024. Median age was 58.5 years (IQR 51.5-67.5), 4 (6.7%) of 60 participants were female, 56 (93.3%) were male. Fifty-six patients (93.3%) were infected with the hepatitis B virus, 37 (61.7%) had liver cirrhosis, 32 (53.3%) had macrovascular invasion, and 8 (13.3%) had extrahepatic metastasis. Fifteen patients (25.0%) were classified as Child-Pugh class B, and 29 (48.3%) had tumors with a maximum diameter exceeding 10 cm. The baseline clinical characteristics of all enrolled patients are summarized in **Table 1**.

**Table 1:** Baseline characteristics of patients.

Baseline characteristics of patients (N = 60)	
Variables	n (%)
Gender	
Female	4 (6.7)
Male	56 (93.3)
Age (years)	
<65	42 (70.0)
≥65	18 (30.0)
HBV infection	
No	4 (6.7)
Yes	56 (93.3)
Cirrhosis	
No	23 (38.3)
Yes	37 (61.7)
ECOG-PS score	
0	23 (38.3)
1	37 (61.7)
ALBI grade	
1	16 (26.7)
2	42 (70.0)
3	2 (3.3)
Child-Pugh classification	
A	45 (75.0)
B	15 (25.0)
BCLC stage	
0-A	12 (20.0)
B	15 (25.0)
C	33 (55.0)
AFP (ng/mL)	
<400	26 (43.3)
≥400	34 (56.7)
TBIL (μmol/L)	
<17.1	19 (31.7)
≥17.1	41 (68.3)
ALT (U/L)	
<35	24 (40.0)
≥35	36 (60.0)
AST (U/L)	
<35	12 (20.0)
≥35	48 (80.0)
Tumor number	
<3	21 (35.0)
≥3	39 (65.0)
Tumor size	
<10cm	31 (51.7)
≥10cm	29 (48.3)
Macrovascular invasion	
No	28 (46.7)
Yes	32 (53.3)
Extrahepatic metastasis	
No	52 (86.7)
Yes	8 (13.3)

HBV, hepatitis B virus; ECOG-PS, Eastern Cooperative Oncology Group Performance Status; ALBI, albumin-bilirubin; BCLC, Barcelona Clinic for Liver Cancer; AFP, alpha-fetoprotein; TBIL, total bilirubin; ALT, alanine aminotransferase; AST, aspartate aminotransferase.

### Efficacy

**Figure 2:**
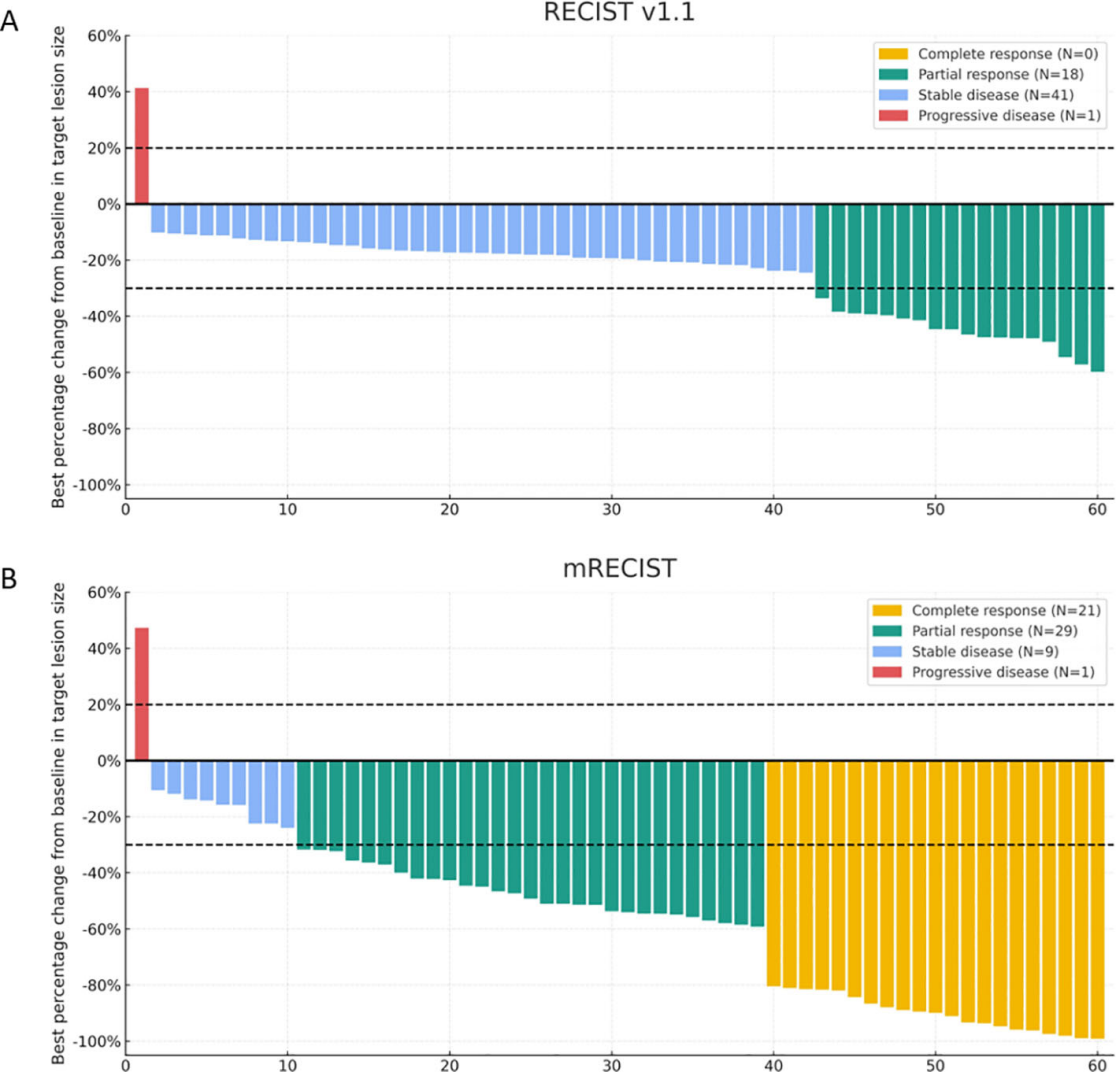
Best percentage change in target lesion size from baseline. Waterfall plots show tumor response based on **(A)** RECIST v1.1 and **(B)** mRECIST criteria.

**Figure 3:**
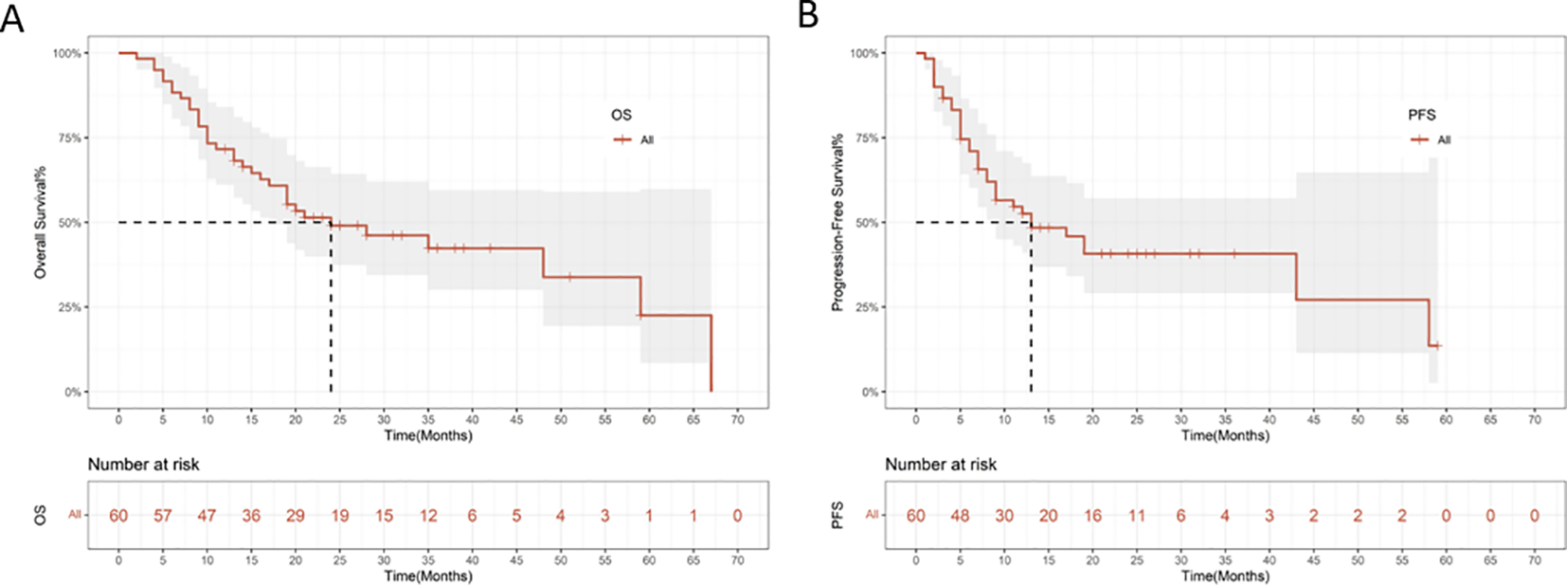
Kaplan-Meier survival curves for overall survival **(A)** and progression-free survival **(B)**.

Tumor response was evaluated according to mRECIST and RECIST v1.1 criteria, and the best overall responses are summarized in **Table 2**. Based on mRECIST, the ORR was 83.3%, and the DCR was 91.7%. According to RECIST v1.1, the ORR was 35.0%, and the DCR was 91.7%. Changes in target lesions are illustrated in **Figures 2A, B**. As of the data cutoff on June 30, 2025, the mOS was 24.0 months (95% CI: 7.659-40.341; **Figure 3A**), and the mPFS was 13.0 months (95% CI: 3.110-22.890; **Figure 3B**).

**Table 2:** Summary of efficacy outcomes.

Summary of efficacy outcomes (N = 60)		
Variables, n (%)	Best overall response (mRECIST)	Best overall response (RECIST v1.1)
CR	16 (26.7)	0 (0.0)
PR	34 (56.7)	21 (35.0)
SD	5 (8.3)	34 (56.7)
PD	5 (8.3)	5 (8.3)
ORR	50 (83.3)	21 (35.0)
DCR	55 (91.7)	55 (91.7)

CR, complete response; PR, partial response; SD, stable disease; PD, progressive disease; ORR, overall response rate; DCR, disease control rate.

Prior to the assessment of PFS, the mean numbers of TACE procedures and cycles of systemic therapy administered were 4.1 and 7.4, respectively.

Among the 60 patients, a total of 53 patients experienced treatment interruption, and 1 patient switched from combination therapy to monotherapy. Upon evaluation, patients subsequently underwent surgical resection (n=8), ablation (n=3), radiotherapy (n=12), or switched to alternative systemic treatment regimens (n=8) following the combination therapy.

### Cox regression analysis

The univariate and multivariate Cox regression analysis of prognostic factors associated with OS was shown in **Table 3**. In univariate analysis, alanine aminotransferase (ALT) (hazard ratio [HR] = 2.55, 95% CI: 1.18-5.53, p = 0.017), macrovascular invasion (MVI) (HR = 2.26, 95% CI: 1.08-4.73, p = 0.030), and extrahepatic metastasis (HR = 2.65, 95% CI: 1.06-6.60, p = 0.036) were significant risk factors for OS. Further multivariate analysis identified macrovascular invasion as an independent prognostic factor for OS (HR = 2.15; 95% CI: 1.02-4.55; P = 0.045).

**Table 3:** Univariate and multivariate Cox regression analysis for OS.

Univariate and Multivariate Cox regression analysis for OS						
Variables	Univariable			Multivariate		
	HR	95%CI	P	HR	95%CI	P
Age(y), (</≥65)	0.98	0.47 ~ 2.03	0.956			
Gender, (female/male)	0.39	0.12 ~ 1.32	0.130			
Cirrhosis, (no/yes)	1.98	0.92 ~ 4.29	0.081			
ECOG-PS score, (0/1)	1.51	0.72 ~ 3.16	0.270			
ALBI grade1	1.00	Reference	–			
ALBI grade2	1.94	0.80 ~ 4.71	0.142			
ALBI grade3	0.00	0.00 ~ Inf	0.997			
Child-Pugh class, (A/B)	0.97	0.42 ~ 2.27	0.953			
BCLC stage 0-A	1.00	Reference				
BCLC stage B	0.96	0.29 ~ 3.16	0.947			
BCLC stage C	2.50	0.93 ~ 6.71	0.070			
AFP(ng/mL), (</≥400)	2.11	0.99 ~ 4.49	0.054			
ALT(U/L), (</≥35)	2.55	1.18 ~ 5.53	**0.017**	2.35	1.05 ~ 5.24	**0.037**
AST(U/L), (</≥35)	2.29	0.80 ~ 6.55	0.122			
Tumor number, (</≥3)	1.46	0.72 ~ 2.98	0.299			
Size(cm), (<10/≥10)	1.38	0.70 ~ 2.75	0.355			
Macrovascular invasion, (no/yes)	2.26	1.08 ~ 4.72	**0.030**	2.15	1.02 ~ 4.55	**0.045**
Extrahepatic metastasis, (no/yes)	2.65	1.06 ~ 6.60	**0.036**	1.67	0.65 ~ 4.29	0.290

ECOG-PS, Eastern Cooperative Oncology Group Performance Status; ALBI, albumin-bilirubin; BCLC, Barcelona Clinic for Liver Cancer; AFP, alpha-fetoprotein; ALT, alanine aminotransferase; AST, aspartate aminotransferase.

Bold values are values with statistical differences (P value<0.05).

The independent prognostic factors associated with PFS was showed **Table 4**. In univariate analysis, gender, ALT, macrovascular invasion, and extrahepatic metastasis were significant factors affecting PFS. Multivariate analysis demonstrated that macrovascular invasion remained an independent prognostic factor for PFS (HR = 2.74; 95% CI: 1.26-5.95; P = 0.011).

**Table 4:** Univariate and multivariate Cox regression analysis for PFS.

Univariate and Multivariate Cox regression analysis for PFS						
Variables	Univariable			Multivariate		
	HR	95%CI	P	HR	95%CI	P
Age(y), (</≥65)	0.77	0.37 ~ 1.64	0.505			
Gender, (female/male)	0.20	0.06 ~ 0.67	**0.009**	0.23	0.04 ~ 1.24	0.087
Cirrhosis, (no/yes)	2.06	0.98 ~ 4.34	0.508			
ECOG-PS score, (0/1)	1.63	0.77 ~ 3.44	0.200			
ALBI grade1	1.00	Reference	–			
ALBI grade2	2.08	0.86 ~ 5.04	0.104			
ALBI grade3	0.00	0.00 ~ Inf	0.997			
Child-Pugh class, (A/B)	0.99	0.43 ~ 2.29	0.983			
BCLC stage 0-A	1.00	Reference				
BCLC stage B	1.31	0.51 ~ 3.35	0.573			
BCLC stage C	1.17	0.47 ~ 2.91	0.731			
AFP(ng/mL), (</≥400)	1.82	0.88 ~ 3.78	0.109			
ALT(U/L), (</≥35)	2.19	1.04 ~ 4.59	**0.039**	1.66	0.77 ~ 3.62	0.199
AST(U/L), (</≥35)	2.56	0.90 ~ 7.31	0.079			
Tumor number, (</≥3)	1.32	0.65 ~ 2.68	0.436			
Size(cm), (<10/≥10)	1.24	0.63 ~ 2.44	0.533			
Macrovascular invasion, (no/yes)	2.63	1.26 ~ 5.49	**0.010**	2.74	1.26 ~5.95	**0.011**
Extrahepatic metastasis, (no/yes)	3.19	1.30 ~ 7.83	**0.011**	1.39	0.41 ~ 4.79	0.599

ECOG-PS, Eastern Cooperative Oncology Group Performance Status; ALBI, albumin-bilirubin; BCLC, Barcelona Clinic for Liver Cancer; AFP, alpha-fetoprotein; ALT, alanine aminotransferase; AST, aspartate aminotransferase.

Bold values are values with statistical differences (P value<0.05).

### Subgroup analysis

**Figure 4:**
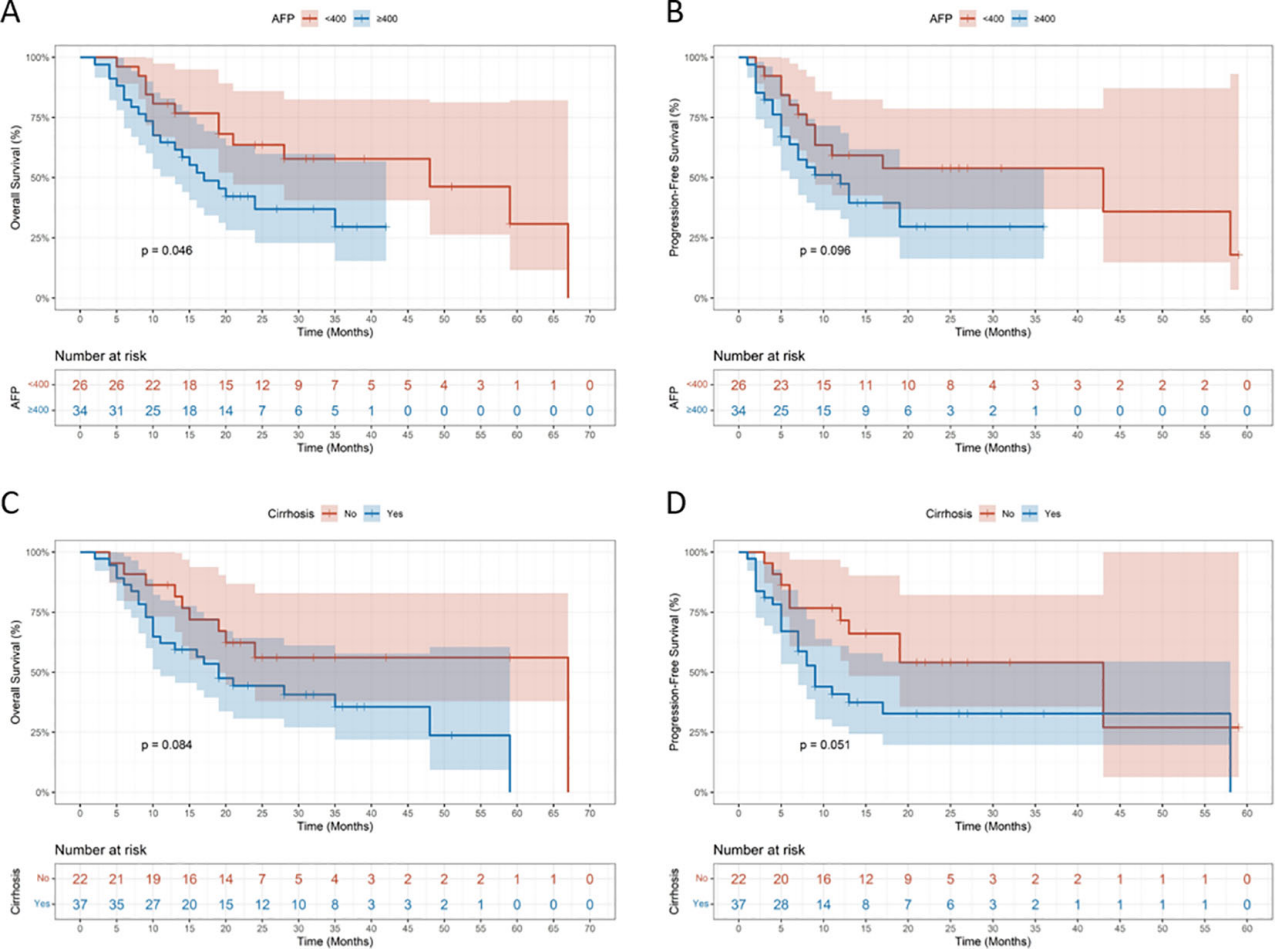
Subgroup Kaplan-Meier survival curves for overall survival and progression-free survival stratified by AFP **(A, B)** and cirrhosis **(C, D)**.

Subgroup analyses were performed according to baseline AFP level and liver cirrhosis status. Patients with AFP ≥ 400 ng/mL had significantly poorer overall survival compared with those with AFP < 400 ng/mL (**Figure 4A**), whereas no significant difference in progression-free survival was observed between the two AFP subgroups (**Figure 4B**). In addition, no significant differences in overall survival (**Figure 4C**) or progression-free survival (**Figure 4D**) were observed between patients with and without liver cirrhosis.

### Safety analysis

A total of 56 patients (93.3%) experienced treatment-related adverse events (tr-AEs) of any grade. The most frequently observed tr-AEs included decreased platelet count (41.7%), proteinuria (35.0%), ALT/AST increased (33.3%), decreased appetite (33.3%), blood bilirubin increased (33.3%), hyperthyroidism (28.3%), abdominal pain (28.3%), fatigue (26.6%), and decreased white blood cell count (25.0%). Fourteen patients (23.3%) developed grade≥3 tr-AEs, with ALT/AST increased being the most common. All tr-AEs were successfully managed, and no treatment-related deaths occurred. Detailed information is provided in **Table 5**.

**Table 5:** Treatment-related adverse events.

Treatment-related adverse events (N = 60)		
Events, n (%)	Any grade	Grade≥3
Patients with treatment-related adverse events	56 (93.3)	14 (23.3)
Decreased platelet count	25 (41.7)	6 (10.0)
Proteinuria	21 (35.0)	0
ALT/AST increased	20 (33.3)	8 (13.3)
Decreased appetite	20 (33.3)	0
Blood bilirubin increased	20 (33.3)	4 (6.7)
Hyperthyroidism	17 (28.3)	0
Abdominal pain	17 (28.3)	1 (1.7)
Fatigue	16 (26.6)	2 (3.3)
Decreased white blood cell count	15 (25.0)	2 (3.3)
Decreased hemoglobin	11 (18.3)	5 (8.3)
Hypoalbuminemia	10 (16.7)	1 (1.7)
Gastrointestinal Hemorrhage	9 (15.0)	0
Fever	5 (8.3)	0
Rash	4 (6.7)	0
Hypothyroidism	3 (5.0)	0

ALT, alanine aminotransferase; AST, aspartate aminotransferase.

In a cohort of 60 patients, two patients discontinued treatment due to adverse events, including one case of gastrointestinal bleeding and one case of rash.

## Discussion

This study evaluated the real-world efficacy and safety of TACE combined with sintilimab (a PD-1 inhibitor) and a bevacizumab biosimilar (an anti-VEGF agent) in 60 patients with unresectable HCC. The key findings revealed a median overall survival (mOS) of 24.0 months, median progression-free survival (mPFS) of 13.0 months, and an objective response rate (ORR) of 83.3% per mRECIST, along with a disease control rate (DCR) of 91.7%. These results demonstrate that the triple combination regimen offers promising efficacy and manageable safety, supporting its potential as a viable therapeutic strategy for patients with uHCC.

With the continuous evolution of HCC treatment paradigms, monotherapy has become increasingly insufficient to meet the clinical demand for prolonged survival and effective disease control (8, 9). In recent years, the integration of locoregional and systemic therapies has emerged as a promising approach to enhance clinical outcomes. In particular, the combination of immunotherapy with anti-angiogenic agents has shown synergistic antitumor activity. However, despite encouraging progress with various combination strategies, the overall prognosis for HCC patients remains suboptimal (10, 11). Therefore, further exploration of optimal combination regimens is warranted to improve therapeutic efficacy and extend survival.

For patients with HCC undergoing TACE monotherapy, the mPFS typically ranges from 7 to 8 months (12, 13). In contrast, combination therapies have demonstrated superior efficacy. The ORIENT-32 trial established sintilimab plus a bevacizumab biosimilar as a first-line regimen for uHCC, showing significantly improved mPFS compared with sorafenib (4.6 vs. 2.8 months; P < 0.0001), though mOS was not reached at the time of reporting (7). Similarly, the IMbrave150 trial reported a 5.8-month improvement in mOS with atezolizumab plus bevacizumab over sorafenib (14). More recently, the CHANCE2211 study demonstrated that TACE combined with camrelizumab and apatinib resulted in significantly longer mOS (24.1 vs. 15.7 months) and mPFS (13.5 vs. 7.7 months) compared with TACE alone (15). Likewise, the phase III LEAP-012 trial showed that TACE plus lenvatinib and pembrolizumab significantly prolonged mPFS versus TACE plus placebo (14.6 vs. 10.0 months; P = 0.0002), with an ORR of 71.3% per mRECIST (16). Consistently, the EMERALD-1 trial revealed that Durvalumab plus bevacizumab plus TACE significantly improved progression-free survival compared with TACE alone (15.0 months vs. 8.2 months; p=0.032) (17). In a nationwide, retrospective, cohort study (n=826), combination therapy (TACE plus PD-[L]1 blockades and molecular targeted treatments) not only improved OS, FPS and ORR more effectively than TACE monotherapy, but also was the most important predictive indicator for OS and PFS (18). These findings collectively highlight the therapeutic advantage of combining TACE with immunotherapy and targeted agents over conventional monotherapy, which has the potential to set a new standard of care.

In this study, we evaluated the therapeutic efficacy and safety of TACE combined with sintilimab and bevacizumab in patients with uHCC. The mOS of 24.0 months in our study was more favorable than that of TACE alone, which was 15.7-19.4 months (4, 18), demonstrating superior outcomes. The mPFS was 13.0 months, which was notably better than the 4.6 months observed in the sintilimab combined with bevacizumab treatment group in the ORIENT-32 study (7). Although our study demonstrated encouraging survival outcomes, these differences arise from indirect cross-study comparisons and are influenced by heterogeneity in patient characteristics, study design, and assessment methods; therefore, the findings should be interpreted with caution and require confirmation in prospective controlled studies. The favorable outcomes of TACE plus sintilimab and bevacizumab biosimilar stem from well-established synergistic mechanisms. First, TACE induces localized tumor ischemia and necrosis, releasing tumor-associated antigens and increasing the expression of PD-L1 in tumor cells, which activates tumor-specific immune response (19, 20). This “*in situ* vaccination” effect is amplified by sintilimab, which blocks PD-1-mediated immune checkpoint inhibition, restoring cytotoxic T-cell function against residual tumor cells (21, 22). Second, TACE upregulates VEGF expression in the hypoxic tumor microenvironment, promoting angiogenesis and immune suppression (e.g., recruitment of regulatory T cells) (3, 23). Bevacizumab counteracts this by inhibiting VEGF, normalizing tumor blood vessels to improve drug delivery and reduce immune suppression (24). Moreover, TACE may be associated with lower intratumoral levels of exhausted effector T cells (CD8^+^/PD-1^+^) and regulatory T cells (CD4^+^/FOXP3^+^), which have the potential to shift the tumor microenvironment from an immunosuppressive to an immune-supportive state and thereby enhance the therapeutic efficacy of PD-L1 inhibitors (24, 25). It should be noted that although VEGF inhibition is a key antitumor mechanism in combination therapy, it can also directly give rise to treatment-related adverse events such as proteinuria. Evidence indicates that these toxicities result from disruption of the physiological roles of VEGF in maintaining the integrity of the glomerular filtration barrier and vascular homeostasis, and thus can be considered manageable “on-target” effects (26, 27). Thus, the combination of TACE, sintilimab, and bevacizumab exhibits complementary mechanisms of action that collectively mitigate tumor progression and confer clinical benefits.

In terms of safety, treatment-related adverse events (tr-AEs) occurred in 93.3% of patients, with decreased platelet count (41.7%), proteinuria (35.0%), and elevated ALT/AST (33.3%) being the most common. Grade ≥ 3 tr-AEs were observed in 23.3% of patients, primarily comprising elevated transaminases. All tr-AEs were effectively managed through a proactive protocol: routine blood monitoring and timely use of platelet-boosting agents for thrombocytopenia; pre-cycle quantitative urine tests and dose adjustment/interruption of bevacizumab for proteinuria; and optimization of TACE embolization combined with hepatoprotective drugs for transaminitis. Crucially, all events were controllable via temporary drug withholding, dose modification, or symptomatic support, and no treatment-related deaths occurred. These findings are consistent with previous reports on TACE combined with immunotherapy and anti-angiogenic agents (15–17), indicating that the triple regimen is generally well-tolerated in clinical practice.

All tr-AEs were effectively managed through a proactive protocol: routine weekly blood monitoring and timely use of platelet-boosting agents for thrombocytopenia; pre-cycle quantitative urine tests and dose adjustment/interruption of bevacizumab for proteinuria; and optimization of TACE embolization combined with hepatoprotective drugs for transaminitis. Crucially, all events were controllable via temporary drug withholding, dose modification, or symptomatic support, and no treatment-related deaths occurred.

Multivariate analysis identified MVI as an independent risk factor for both OS (HR = 2.15, P = 0.045) and PFS (HR = 2.74, P = 0.011) in our cohort—consistent with Qin et al.’s findings (HR = 3.13 for OS, HR = 2.55 for PFS) (28) and EMERALD-1’s subgroup analysis, which showed poorer progression-free survival in patients with MVI (HR = 1.12 vs. 0.73 for non-MVI patients) (17). MVI is a well-recognized marker of aggressive tumor biology, as it indicates tumor cell invasion into major vascular structures and increased risk of extrahepatic metastasis. Multivariate analysis in previous study reported that MVI was significantly associated with tumor necrosis by TACE (*P* <0 .02), and the strongest factor for recurrence-free survival rate within 2 years (*P* = 0.002) (29). These observations imply that prevention of macrovascular invasion may help improve clinical outcomes.

In the present study, patients with baseline AFP ≥ 400 ng/mL had significantly poorer overall survival compared with those with AFP < 400 ng/mL, which is consistent with previous reports (30), suggesting that elevated AFP is closely associated with unfavorable long-term prognosis in patients with hepatocellular carcinoma. However, no significant difference in progression-free survival was observed between patients with high and low AFP levels, indicating that AFP may have a limited impact on short-term disease progression. In contrast, liver cirrhosis was not significantly associated with either overall survival or progression-free survival, demonstrating that prognosis in this combination treatment setting may be driven more by tumor-related factors than by underlying cirrhotic status.

Several limitations of this study should be acknowledged. First, the retrospective, single-center design with a modest sample size may introduce selection bias. Second, the absence of a control group limits the ability to directly compare efficacy with other regimens, and thus the conclusions are drawn from indirect comparisons with historical data. Third, although the first-line treatment was standardized, subsequent therapies after progression varied among patients, which may have confounded the survival analysis. Future large-scale, multicenter, prospective randomized trials are warranted to validate these findings and further establish the generalizability of this combination strategy.

## Conclusion

In conclusion, our study suggests that the combination of TACE with sintilimab and bevacizumab provides a safe and effective therapeutic option for patients with HCC, showing considerable promise in enhancing treatment efficacy and improving clinical outcomes.

## Data Availability

The original contributions presented in the study are included in the article/supplementary material. Further inquiries can be directed to the corresponding author.
